# First report of *Candida auris* in Romania: clinical and molecular aspects

**DOI:** 10.1186/s13756-023-01297-x

**Published:** 2023-09-07

**Authors:** Adriana Mihaela Stanciu, Dragoș Florea, Marius Surleac, Simona Paraschiv, Dan Oțelea, Daniela Tălăpan, Gabriel Adrian Popescu

**Affiliations:** 1 “Carol Davila” University of Medicine and Pharmacy, Dionisie Lupu Street no 37, Bucharest, 050474, Romania; 2 “Prof. Dr. Matei Bals” National Institute of Infectious Diseases, Calistrat Grozovici Street no 1, Bucharest, 021105 Romania

**Keywords:** *Candida auris*, Healthcare-associated infections

## Abstract

The emerging opportunistic fungal pathogen *Candida auris* raises significant concerns for public health due to its outbreak potential, the associated high mortality, increased resistance to antifungals, challenging identification to species level, since commonly used diagnostic methods can confuse this fungus with other *Candida spp*. The present outbreak report describes probably some of the first *Candida auris* cases in Romania, providing clinical and epidemiological data, and also whole genome sequencing data. The cases were identified in three hospitals in Bucharest during the first eight months of 2022.

## Introduction

Described for the first time in 2009, the emergent fungus *Candida auris* has been associated with nosocomial outbreaks and has raised significant problems for healthcare facilities due particular characteristics: prolonged persistence on patients’ skin and mucosal surfaces as well as in the hospital environment, high transmissibility, ability to cause severe systemic infections in vulnerable patients and an increased resistance to routinely used antifungal drugs [1–4]. The first hospital outbreak in Europe was reported in 2016 in a cardio-thoracic center in London, UK, with 50 *C.auris* cases identified between April 2015 and July 2016 [5]. This was followed between April 2016 and January 2017 by a second, more extensive outbreak in Spain which involved 140 patients with colonization and 41 with invasive bloodstream infections [6].The European Centre for Disease Prevention and Control (ECDC) has issued three reports between 2018 and 2022 providing updated information about the progress of the laboratory diagnostic capacities in European countries, the number of cases, and control efforts to increase awareness regarding this pathogen and limit its rapid spread across the continent [7].

The first *Candida auris* isolates in Romania were identified at the beginning of 2022 in several hospitals in Bucharest which were adequately equipped for proper identification for this recently described fungus. Accumulating data raise concerns regarding a possibility of an epidemic outbreak caused by this pathogen in Bucharest. The present report describes clinical and molecular features of the first cases of *Candida auris* infection and colonization in Romania. No previous *Candida auris* isolates were reported in Romania either because the fungus hadn’t yet emerged in our country or, possibly, because reliable methods of identification were not available at a national level.

## Materials and methods

A retrospective observational study was conducted in three hospitals in Bucharest. All patients with *Candida auris* isolates identified from January 2022 to August 2022 were included in this study. The aforementioned hospitals are tertiary care facilities with 430 (hospital 1), 987 (hospital 2) and 1620 (hospital 3) beds, all equipped with at least one intensive care unit and having a generally high consumption of second- and third-line antibiotics [8]. *Candida auris* was also identified in several other hospitals in Bucharest but the isolates were not available for analysis.

The data collected included demographics, the primary diagnosis, comorbidities, risk factors for *C. auris*, whether the *C. auris* identification was considered an infection or colonization and outcome.

The *C. auris* identification was regarded as an infection if the specimen was collected from a normally sterile site or if the attending physician considered it to be an infection based on patient’s symptoms and laboratory findings. In the case of patients with multiple isolation sites, if at least one of the positive samples was interpreted as an infection we considered the respective patients as having a *C. auris* infection, even if they also had simultaneous colonization sites.

The *C. auris* isolates were identified using a MALDI-TOF MS (Bruker Daltonics GmbH & KG, Bremen, Germany) system and antifungal susceptibility was determined using broth microdilution Micronaut – AM plates (Bruker Daltonics GmbH & KG, Bremen, Germany). Antifungal susceptibility data are available for 18 of the isolates. The results were interpreted using the CDC tentative MIC breakpoints [9].

Due to limited resources, whole genome sequencing (WGS) was only performed for sixteen of the isolates, selected as follows: all isolates from hospitals 1 and 2 and from hospital 3, the first 9 that were retrieved by the Infection Prevention and Control team for this purpose, when it became clear they were confronting a serious epidemiological situation.

The sixteen *C.auris* isolates were sequenced using Illumina MiSeq and NextSeq 550 Next Generation Sequencing platforms (NGS). DNA extraction was performed with Ultra clean microbial kit (Qiagen) and DNA samples were further processed for NGS by using Illumina DNA Prep with enrichment and following the manufacturer’s recommendations.

The generated reads have been polished, trimmed and assembled *de novo* using the shovill pipeline [10]. The resulted contigs were further used to perform a BLAST reference sequence search. The raw reads have been mapped on the best BLAST hits references, using Geneious Prime 2023.0.1 software [11].

The raw reads as well as the resulted consensus sequences for each of the 16 sequenced strains, were further subject of clade classification, by mapping them onto clade specific sequences (CSS), as previously described by others [11, 12]. The CSSs were downloaded from the NCBI database.

The clade classification was performed as follows: first, the raw reads for each sample were mapped onto all CSS sequences; secondly, the consensus sequences from each chromosome in each of the sequenced strains were also used as templates for CSS sequence mapping.

Variant calling was performed by aligning the Illumina raw reads on the GCA_019039555.1 GenBank reference genome using snippy version 4.5.1 (https://github.com/tseemann/snippy) with default options, deploying BWA, FreeBayes and SAMtools tools.

The sixteen isolates raw data has been deposited to SRA NCBI database under BioProject number PRJNA991119 (https://www.ncbi.nlm.nih.gov/sra/PRJNA991119).

## Results

### Patient characteristics and risk factors

During the study period *Candida auris* was isolated from 40 patients, of which 32 (80%) were men. The mean age was 63.3 years (19–94 years). Most of the patients were critically ill, with 24 cases (60%) being diagnosed in intensive care units. Half of the patients had as a primary diagnosis a severe infection with a pathogen other than *Candida auris*, 8 (20%) had been admitted for stroke and the rest had other diagnoses. Nine patients (22.5%) were diagnosed with COVID-19 during the current hospital admission or in the prior 14 days. All but two patients had underlying comorbidities including cardiovascular disease (25), diabetes mellitus (12), chronic kidney disease (6), liver disease (4), malignancy (3), chronic lung disease (2) and HIV infection (2) (Table 1).

**Table 1:** *Candida auris* cases in the three hospitals included in the study

	Hospital 1	Hospital 2	Hospital 3	Total
**Demographics**				
Mean age (years)	66.5	50.8	65.3	63.3
Gender (% of men)	100.00%	60.00%	81.80%	80.00%
**Comorbidities**				
cardiovascular disease	1	2	22	25
diabetes mellitus	0	1	11	12
CKD	0	2	4	6
liver disease	0	0	4	4
malignancy	1	0	2	3
chronic lung disease	0	0	2	2
HIV	0	0	2	2
**Risk factors for** ***C. auris*** **presence**				
mean days before first isolation	25.5	23.2	34.7	32.8
patients with prior ICU admission	1	4	26	31
broad spectrum antibiotics before isolation	2	5	33	40
antifungals before isolation	1	2	24	27
COVID-19	1	0	8	9
Total patients with infection*				
bloodstream infection	0	4	11	15
urinary	0	3	7	10
hospital-acquired pneumonia	0	0	5	5
infected bedsore	0	0	1	1
patients with multiple-site isolation	0	3	3	6
**Total patients with colonization**				
urine	2	0	3	5
central line	0	1	1	2
upper respiratory tract	0	0	1	1
**Undetermined**	0	0	7	7
upper respiratory tract	0	0	5	5
urine	0	0	2	2
All-cause mortality for infected patients	0	1 (25%)	17 (81%)	18 (72%)
All cause mortality for colonized patients	1 (50%)	1 (100%)	2 (40%)	4 (50%)
All cause mortality for undetermined cases	0	0	4 (57%)	4 (57%)
Total cases	2	5	33	40

*The total number of isolation sites is greater than the number of patients because six patients had two isolation sites of infection.

Among the identified *C. auris* cases, 25 were infections, 8 were asymptomatic carriages and 7 cases were not clearly differentiated as infection or colonization (Table 1). Most of the infections were bloodstream infections (60%), followed by urinary infections (20%), hospital acquired pneumonia (16%) and one infected pressure sore. Nine patients (22,5%) had multiple-site *Candida auris* identification, out of which 6 were infections and 3 were undetermined.

Three colonization sites were identified: urine (5 patients), central venous catheter (2 patients) and one patient had upper respiratory tract carriage. Skin colonization can be presumed to have been present as well, but *Candida auris* colonization screening hadn’t yet been implemented in these hospitals during the study period.

The risk factors for *Candida auris* infection or colonization were similar to those associated with other *Candida* species infections: prolonged hospitalization (mean number of days before first *Candida auris* isolation was 32.8 days, ranging between 0 and 81 days, SD = 18.3 days), ICU admission (31/40 patients (77.5%) had been admitted in an ICU prior to *Candida auris* identification, with a mean of 32.1 days of ICU stay), use of broad spectrum antibiotics (all patients had received broad spectrum antibiotics prior to *C. auris* diagnosis, including carbapenems for 77.5% of them), concomitant carbapenem-resistant *Enterobacterales* colonization (70%), antifungal treatment (67.5%), central venous catheterization (80%) and other invasive medical procedures. The crude mortality rate for patients with *Candida auris* infection was 72% (18/25 patients).

### *Candida auris* identification, antifungal susceptibility and clade assessment

Using the CDC tentative MIC breakpoints [9] for the susceptibility interpretation, we found that all isolates were resistant to fluconazole (with a MIC ranging between 32 µg/mL and 128 µg/mL), 4 (22.2%) were resistant to amphotericin B, and all 18 isolates were susceptible to anidulafungin and micafungin (Table 2).

**Table 2:** Antifungal susceptibility of the C. auris isolates (MICs in mg/L)

	AMB	FLU	VOR	POS	ITR	MFG	AFG
Breakpoints*	≥ 2	≥ 32	-	-	-	≥ 4	≥ 4
Pt. 1	1	> 128	0.5	0.03	0.25	0.125	0.125
Pt. 2	4	128	4	8	4	0.125	0.25
Pt. 3	0.5	128	0.25	<=0.0078	<=0.0312	0.0312	0.0312
Pt. 4	0.5	64	0.25	<=0.0078	0.0312	0.0156	0.0156
Pt. 5	0.5	64	0.25	0.0156	<=0.0312	0.0625	0.0625
Pt. 6	0.5	> 128	0.5	0.0156	<=0.0312	0.0312	0.0625
Pt. 7	1	> 128	0.5	0.0156	0.0625	0.0625	0.0625
Pt. 8	1	> 128	0.5	0.0156	0.0625	0.0312	0.0312
Pt. 9	0.5	> 128	0.5	0.0156	<=0.0312	0.0156	0.125
Pt. 10	0.5	128	0.25	<=0.0078	<=0.0312	0.0312	0.0625
Pt. 11	0.5	64	0.5	0.0156	<=0.0312	0.0625	0.0625
Pt. 12	0.5	> 128	0.5	0.0156	<=0.0312	0.0625	0.0625
Pt. 13	0.5	>128	0.5	0.0156	<=0.0312	0.0625	0.0625
Pt. 14	0.5	> 128	0.5	0.0156	<=0.0312	0.0625	0.0625
Pt. 15	0.5	>128	0.5	0.0156	<=0.0312	0.0156	0.0625
Pt. 16	8	32	1	-	-	0.12	-
Pt. 17	>=16	32	1	-	-	0.12	-
Pt. 18	>=16	32	1	-	-	0.12	0.25
Range	0.5–16	32–128	0.25-4	0.0078-8	0.0312-4	0.0156–0.125	0.0156-0.25

*Abbreviations: AMB – Amphotericin B, FLU – Fluconazole, VOR – Voriconazole, POS – Posaconazole, ITR – Itraconazole, MFG – Micafungin, AFG – Anidulafungin.*

*CDC tentative breakpoints [9]

NGS results were used for clade classification. CSS1 was chosen which was well covered: between 100k and 350k raw reads mapped with more than 98% identity. These results indicate that all studied Romanian isolates belong to the Southern Asian clade (clade I). Single nucleotide polymorphism revealed that almost all but two isolates are highly correlated (Pearson coefficients of 0.98).

## Discussion

*Candida auris* is an emerging multidrug-resistant fungus with intra- and interhospital transmission that raises significant concerns for healthcare facilities. The current report analyses the first identified cases of infection and colonization with this fungus in three hospitals in Bucharest.

Several similarities were found between the patients’ profiles from the current study and those identified by other authors. Most *Candida auris* infections or carriages were diagnosed in men and generally in severely ill patients [13]. Comorbidities associated with *Candida auris* identification include sepsis, diabetes mellitus, malignancies, HIV infection, cardiovascular diseases, chronic kidney disease and chronic pulmonary diseases [13–15]. Prolonged hospitalization has been linked to an increased risk of acquiring a *Candida auris* infection with a time period of 10 to 50 days from admission to diagnosis, and so did ICU admission, the presence of central venous catheters and exposure to broad-spectrum antibiotics and antifungals [16–19]. Although *Candida auris* has been associated with the surge of COVID-19 hospitalizations during 2021 [20], a meta-analysis in 2022 found a lower prevalence of *C. auris* infections among COVID patients than in the pre-COVID era [21].

The present study identified an all-cause mortality for patients with *Candida auris* infection slightly higher than previously described, which is between 30% and 60%, but we have to consider the fact that there is a high variability among different studies. Moreover, it was usually difficult to determine the extent to which the mortality is attributable to *Candida auris* infection or the other severe conditions generally present in these patients [13, 16].

Since all *Candida auris* isolates from this study belong to the Southern Asian clade (clade I), and (single-nucleotide polymorphism) analysis showed that all but two isolates are highly similar, a clonal dissemination in and between the three hospitals included in the study is highly probable, similar to the outbreak described in northern Italy [22]. However, since two isolates from the current study were not similar to the identified clone, the hypothesis of multiple introduction events is also plausible. Further studies with a higher number of isolates from more settings with a denser sampling strategy, including strains from colonized individuals, and additional molecular analyses are required to investigate this possibility.

*Candida auris*’ resistance to fluconazole is known to be high (around 90% in most studies). The isolates included in this study followed the same pattern and were all classified as resistant according to the CDC tentative MIC breakpoints [9, 23]. Susceptibility to amphotericin B and echinocandins was similar to those previously described in different studies [19, 23, 24].

The present study has limitations. First, being a retrospective analysis, the information regarding *Candida auris* infections or colonization was sometimes incomplete or could not be interpreted without the necessary context. Second, not all isolates were available for antifungal susceptibility testing or whole genome sequencing.

## Conclusion

This communication emphasizes three important issues: the potential of *C. auris* to become a public health problem in Romania, the need for active surveillance of this fungus and the consequent requirement for adapted infection control strategies, including improved diagnostic tools throughout the country.

## Data Availability

The data that support the findings of this study are available from the corresponding author upon reasonable request. NGS data generated in this study has been uploaded in NCBI Sequence Read Archive (SRA) available at https://www.ncbi.nlm.nih.gov/sra/PRJNA991119.

## Abbreviations

*C. auris*: *Candida auris*
CKD: chronic kidney disease
ICU: intensive care unit
MALDI-TOF MS: Matrix-Assisted Laser Desorption/Ionization Time-of-Flight Mass Spectrometry
NGS: Next-Generation Sequencing

## References

[1] Chowdhary A, Jain K, Chauhan N. Candida auris Genetics and Emergence. Annu Rev Microbiol. 2023 5. 10.1146/annurev-micro-032521-015858 PMC12962553 37406342

[2] Bravo Ruiz G, Lorenz A (2021). What do we know about the biology of the emerging fungal pathogen of humans Candida auris?. Microbiol Res.

[3] Understanding C. auris Transmission with the Healthcare Environment [Internet]. ASM.org. [cited 2023 Jan 12]. Available from: https://asm.org/Press-Releases/2019/June/Understanding-C-auris-Transmission-with-the-He-1.

[4] General Information about Candida. auris | Candida auris | Fungal Diseases | CDC [Internet]. 2019 [cited 2023 Jan 12]. Available from: https://www.cdc.gov/fungal/candida-auris/candida-auris-qanda.html.

[5] Schelenz S, Hagen F, Rhodes JL, Abdolrasouli A, Chowdhary A, Hall A (2016). First hospital outbreak of the globally emerging Candida auris in a european hospital. Antimicrob Resist Infect Control.

[6] Ruiz-Gaitán A, Moret AM, Tasias-Pitarch M, Aleixandre-López AI, Martínez-Morel H, Calabuig E (2018). An outbreak due to Candida auris with prolonged colonisation and candidaemia in a tertiary care european hospital. Mycoses.

[7] Kohlenberg A, Monnet DL, Plachouras D. Group C auris survey collaborative. Increasing number of cases and outbreaks caused by Candida auris in the EU/EEA, 2020 to 2021. Eurosurveillance. 202217;27:2200846. 10.2807/1560-7917.ES.2022.27.46.2200846 PMC9673237 36398575

[8] Consumul de antibiotice., Rezistența microbiană și Infecții Asociate Asistenței Medicale în România, 2020:84. available at https://www.cnscbt.ro/.

[9] Antifungal Susceptibility Testing and Interpretation. | Candida auris | Fungal Diseases | CDC [Internet]. 2022 [cited 2023 Mar 4]. Available from: https://www.cdc.gov/fungal/candida-auris/c-auris-antifungal.html.

[10] https://github.com/tseemann/shovill.

[11] Altschul SF, Gish W, Miller W, Myers EW, Lipman DJ (1990). Basic local alignment search tool. J Mol Biol.

[12] Narayanan A, Selvakumar P, Siddharthan R, Sanyal K (2022). ClaID: a Rapid Method of Clade-Level Identification of the Multidrug Resistant Human Fungal Pathogen Candida auris. Microbiol Spectr.

[13] Osei Sekyere J (2018). Candida auris: a systematic review and meta-analysis of current updates on an emerging multidrug-resistant pathogen. MicrobiologyOpen.

[14] Chowdhary A, Sharma C, Duggal S, Agarwal K, Prakash A, Singh PK (2013). New clonal strain of Candida auris, Delhi, India. Emerg Infect Dis.

[15] Morales-López SE, Parra-Giraldo CM, Ceballos-Garzón A, Martínez HP, Rodríguez GJ, Álvarez-Moreno CA (2017). Invasive infections with multidrug-resistant yeast Candida auris, Colombia. Emerg Infect Dis.

[16] Chowdhary A, Sharma C, Meis JF (2017). Candida auris: a rapidly emerging cause of hospital-acquired multidrug-resistant fungal infections globally. PLoS Pathog.

[17] Calvo B, Melo ASA, Perozo-Mena A, Hernandez M, Francisco EC, Hagen F (2016). First report of Candida auris in America: clinical and microbiological aspects of 18 episodes of candidemia. J Infect.

[18] Forsberg K, Woodworth K, Walters M, Berkow EL, Jackson B, Chiller T (2019). Candida auris: the recent emergence of a multidrug-resistant fungal pathogen. Med Mycol.

[19] Vallabhaneni S, Kallen A, Tsay S, Chow N, Welsh R, Kerins J (2017). Investigation of the First Seven reported cases of Candida auris, a globally emerging invasive, multidrug-resistant Fungus-United States, May 2013-August 2016. Am J Transplant off J Am Soc Transplant Am Soc Transpl Surg.

[20] Biran R, Cohen R, Finn T, Brosh-Nissimov T, Rahav G, Yahav D et al. Nationwide Outbreak of Candida auris Infections Driven by COVID-19 Hospitalizations, Israel, 2021–2022 - Volume 29, Number 7—July 2023 - Emerging Infectious Diseases journal - CDC. [cited 2023 Aug 17]; Available from: https://wwwnc.cdc.gov/eid/article/29/7/22-1888_article. 10.3201/eid2907.221888 PMC10310389 37347492

[21] Vaseghi N, Sharifisooraki J, Khodadadi H, Nami S, Safari F, Ahangarkani F (2022). Global prevalence and subgroup analyses of coronavirus disease (COVID-19) associated Candida auris infections (CACa): a systematic review and meta‐analysis. Mycoses.

[22] Rapid risk assessment. : Candida auris outbreak in healthcare facilities in northern Italy, 2019–2021 [Internet]. 2022 [cited 2023 Mar 13]. Available from: https://www.ecdc.europa.eu/en/publications-data/rapid-risk-assessment-candida-auris-outbreak-healthcare-facilities-northern-italy.

[23] Lockhart SR, Etienne KA, Vallabhaneni S, Farooqi J, Chowdhary A, Govender NP (2017). Simultaneous emergence of Multidrug-Resistant Candida auris on 3 continents confirmed by whole-genome sequencing and epidemiological analyses. Clin Infect Dis off Publ Infect Dis Soc Am.

[24] Kathuria S, Singh PK, Sharma C, Prakash A, Masih A, Kumar A (2015). Multidrug-resistant Candida auris Misidentified as Candida haemulonii: characterization by Matrix-Assisted laser desorption ionization-time of Flight Mass Spectrometry and DNA sequencing and its Antifungal Susceptibility Profile variability by Vitek 2, CLSI Broth Microdilution, and Etest Method. J Clin Microbiol.

